# Supplementary material for: Dietary reference intake for military operations: a scoping review
Source: PeerJ. 2024 Nov 4;12:e18353. doi: 10.7717/peerj.18353 (PMC11542563; doi:10.7717/peerj.18353)
Supplement: Supplemental Information 2 [file peerj-12-18353-s002.docx]

**Supplementary Materials**

**Table S1. List of Dietary Reference Intakes (DRI) indicators and their abbreviations for civilians and military**

|  | Civilian | | Military | |
| --- | --- | --- | --- | --- |
| Australia | Nutrient Reference Values; NRV | For Energy ・Estimated Energy Requirement; EER For Nutrients ・Estimated Average Requirement; EAR ・Recommended Dietary Intake; RDI ・Adequate Intake; AI ・Upper Level of Intake; UL ・Acceptable Macronutrient Distribution Range; AMDR ・Suggested Dietary Target; SDT | Military Nutrient Reference Value; MNRV | For Energy and Nutrients  ・Military Recommended Dietary Intake; MRDI  ・Military Estimated Average Requirement; MEAR |
| UK | Dietary Reference Value; DRV | For Energy ・Estimated Average Requirement; EAR For Nutrients ・Estimated Average Requirement; EAR ・Reference Nutrient Intake; RNI ・Safe Intakes ・Lower Reference Nutrient Intake; LRNI ・Dietary Reference Value; DRV ・Population average | Military Dietary Reference Value; Military DRV | For Energy and Nutrients  ・Military Dietary Reference Value; Military DRV |
| USA | Dietary Reference Intakes; DRI^+^ | For Energy ・Estimated Energy Requirement; EER For Nutrients ・Estimated Average Requirement; EAR ・Recommended Dietary Allowance; RDA ・Adequate Intake; AI ・Acceptable Macronutrient Distribution Range; AMDR ・Tolerable Upper Intake Level; UL  ・Chronic Disease Risk Reduction Intake; CDRR | Military Dietary Reference Intake; MDRI^+^ | For Energy and Nutrients  ・Military Dietary Reference Intake; MDRI  ・Nutritional Standards for Operational Ration; NSOR  ・Nutritional Standards for Restricted Ration; NSRR |
| NATO | Nutrient Reference Values; NRVs | For Energy ・Estimated Energy Requirement; EER For Nutrients ・Estimated Average Requirement; EAR ・Recommended Dietary Intake; RDI ・Adequate Intake; AI ・Upper Level of Intake; UL ・Acceptable Macronutrient Distribution Range; AMDR ・Suggested Dietary Target; SDT | Nutrient Intake Value; NIV | For Energy and Nutrients ・Average Nutrient Requirement; ANR ・Acceptable macronutrient distribution range; AMDR ・Adequate Intake; AI ・Individual Nutrient Level 97.5; INL97.5 |
| The DRI in the USA was the “Recommended Dietary Allowances (RDA)” from 1941 to 1997, but since then it has become the “Dietary Reference Intakes (DRI)” in the USA and Canada. In 1985, USA’s DRI for Military Operations was formulated as “Military Recommended Dietary Allowances (MRDA)”. | | | | |

**Table S2. Physical activity level (PAL) categories and energy requirement in Dietary Reference Intakes (DRI) for civilians and military (/day)**

| DRI | Age (years) | Category | PAL (Range) | Males | | | | | Females | | | | |
| --- | --- | --- | --- | --- | --- | --- | --- | --- | --- | --- | --- | --- | --- |
|  |  |  |  | Reference body size | | | Energy requirements | | Reference body size | | | Energy requirements | |
|  |  |  |  | Heigh (cm) | Weight (kg) | BMI (kg/m^2^) | (MJ) | (kcal) | Heigh (cm) | Weight (kg) | BMI (kg/m^2^) | (MJ) | (kcal) |
| Civilian of Australia, 2006 | 16 | Bed rest-Vigorous activity | 1.2–2.2 | 174.0 | 60.9 | 20.1 | 8.9–16.2 | 2127–3513 | 163.0 | 53.9 | 20.3 | 7.2–13.1 | 1721–3131 |
|  | 17 |  |  | 175.0 | 64.6 | 21.1 | 9.2–16.7 | 2199–3991 | 163.0 | 55.1 | 20.7 | 7.2–13.2 | 1721–3155 |
|  | 18 |  |  | 176.0 | 67.2 | 21.7 | 9.4–17.1 | 2247–4087 | 163.0 | 56.2 | 21.2 | 7.3–13.3 | 1745–3179 |
|  | 19–30 | Bed rest | 1.2 | 160–200 | 56.3–88 | 22.0 | 7.7–8.4 | 1840–2008 | 150–190 | 49.5–79.4 | 22.0 | 6.1–8.4 | 1458–2008 |
|  |  | Very sedentary | 1.4 |  |  |  | 9.0–11.8 | 2151–2820 |  |  |  | 7.1–9.7 | 1697–2318 |
|  |  | Light activity | 1.6 |  |  |  | 10.3–13.5 | 2462–3227 |  |  |  | 8.2–11.1 | 1960–2653 |
|  |  | Moderate activity | 1.8 |  |  |  | 11.6–15.2 | 2772–3633 |  |  |  | 9.2–12.5 | 2199–2988 |
|  |  | Heavy activity | 2.0 |  |  |  | 12.9–16.9 | 3083–4039 |  |  |  | 10.2–13.9 | 2438–3322 |
|  |  | Vigorous activity | 2.2 |  |  |  | 14.2–18.6 | 3394–4446 |  |  |  | 11.2–15.3 | 2677–3657 |
|  | 31–50 | Bed rest | 1.2 | 160–200 | 56.3–88 | 22.0 | 7.6–9.5 | 1816–2271 | 150–190 | 49.5–79.4 | 22.0 | 6.3–7.5 | 1506–1793 |
|  |  | Very sedentary | 1.4 |  |  |  | 8.9–11.0 | 2127–2629 |  |  |  | 7.3–8.3 | 1745–2079 |
|  |  | Light activity | 1.6 |  |  |  | 10.2–12.6 | 2438–3011 |  |  |  | 8.4–10.0 | 2008–2390 |
|  |  | Moderate activity | 1.8 |  |  |  | 11.4–14.2 | 2725–3394 |  |  |  | 9.4–11.2 | 2247–2677 |
|  |  | Heavy activity | 2.0 |  |  |  | 12.7–15.8 | 3035–3776 |  |  |  | 10.4–12.5 | 2486–2988 |
|  |  | Vigorous activity | 2.2 |  |  |  | 14.0–17.3 | 3346–4135 |  |  |  | 11.5–13.7 | 2749–3274 |
|  | 51–70 | Bed rest | 1.2 | 160–200 | 56.3–88 | 22.0 | 7.0–8.8 | 1673–2103 | 150–190 | 49.5–79.4 | 22.0 | 6.0–7.2 | 1434–1721 |
|  |  | Very sedentary | 1.4 |  |  |  | 8.2–10.2 | 1960–2438 |  |  |  | 6.9–8.4 | 1649–2008 |
|  |  | Light activity | 1.6 |  |  |  | 9.3–11.7 | 2223–2796 |  |  |  | 7.9–9.6 | 1888–2294 |
|  |  | Moderate activity | 1.8 |  |  |  | 10.4–13.2 | 2486–3155 |  |  |  | 8.9–10.8 | 2127–2581 |
|  |  | Heavy activity | 2.0 |  |  |  | 11.5–14.7 | 2749–3513 |  |  |  | 9.8–12.0 | 2342–2868 |
|  |  | Vigorous activity | 2.2 |  |  |  | 12.7–16.1 | 3035–3848 |  |  |  | 10.9–13.2 | 2605–3155 |
| Military of Australia, 2009 | 17–18 | Low physical activity | 1.6 | 180.0 | 78.0 | 24.1 | 12.5 | 2988 | 180.0 |  | 24.1 | 9.0 | 2151 |
|  |  | Moderately active | 1.8 |  |  |  | 14.5 | 3466 |  |  |  | 10.5 | 2510 |
|  |  | Very active | 2.0 |  |  |  | 16.5 | 3944 |  |  |  | 12.0 | 2868 |
|  |  | Extreme activity | 2.4 |  |  |  | 19.5 | 4661 |  |  |  | 13.5 | 3227 |
|  | 19–30 | Low physical activity | 1.6 |  |  |  | 12.0 | 2868 |  |  |  | 8.5 | 2032 |
|  |  | Moderately active | 1.8 |  |  |  | 14.0 | 3346 |  |  |  | 10.0 | 2390 |
|  |  | Very active | 2.0 |  |  |  | 16.0 | 3824 |  |  |  | 11.5 | 2749 |
|  |  | Extreme activity | 2.4 |  |  |  | 19.0 | 4541 |  |  |  | 13.0 | 3107 |
|  |  | Believed to approach the limit of human endurance^**^ | 3.2 |  |  |  | 25.0 | 5975 | ― | ― | ― | ― | ― |
| Civilian of UK, 2011 | 16 | 25th percentile (Less active) | 1.68 | 173.0 | 60.2 | 20.1 | 11.9 | 2844 | 163.0 | 55.3 | 20.8 | 9.7 | 2318 |
|  |  | Median (Population) | 1.75 |  |  |  | 12.4 | 2964 |  |  |  | 10.1 | 2414 |
|  |  | 75th percentile (More active) | 1.86 |  |  |  | 13.2 | 3155 |  |  |  | 10.8 | 2581 |
|  | 17 | 25th percentile (Less active) | 1.68 | 175.0 | 64.0 | 20.9 | 12.3 | 2940 | 163.0 | 57.0 | 21.5 | 9.8 | 2342 |
|  |  | Median (Population) | 1.75 |  |  |  | 12.9 | 3083 |  |  |  | 10.3 | 2462 |
|  |  | 75th percentile (More active) | 1.86 |  |  |  | 13.7 | 3274 |  |  |  | 10.9 | 2605 |
|  | 18 | 25th percentile (Less active) | 1.68 | 176.0 | 66.2 | 21.4 | 12.6 | 3011 | 163.0 | 57.2 | 21.5 | 9.9 | 2366 |
|  |  | Median (Population) | 1.75 |  |  |  | 13.2 | 3155 |  |  |  | 10.3 | 2462 |
|  |  | 75th percentile (More active) | 1.86 |  |  |  | 14.0 | 3346 |  |  |  | 11.0 | 2629 |
|  | 19–24 | 25th percentile (Less active) | 1.49 | 178.0 | 71.5 | 22.6 | 10.6 | 2533 | 163.0 | 59.9 | 22.5 | 8.4 | 2008 |
|  |  | Median (Population) | 1.63 |  |  |  | 11.6 | 2772 |  |  |  | 9.1 | 2175 |
|  |  | 75th percentile (More active) | 1.78 |  |  |  | 12.6 | 3011 |  |  |  | 10.0 | 2390 |
|  | 25–35 | 25th percentile (Less active) | 1.49 | 178.0 | 71.0 | 22.4 | 10.5 | 2510 | 163.0 | 59.7 | 22.5 | 8.3 | 1984 |
|  |  | Median (Population) | 1.63 |  |  |  | 11.5 | 2749 |  |  |  | 9.1 | 2175 |
|  |  | 75th percentile (More active) | 1.78 |  |  |  | 12.6 | 3011 |  |  |  | 10.0 | 2390 |
|  | 35–44 | 25th percentile (Less active) | 1.49 | 176.0 | 69.7 | 22.5 | 10.0 | 2390 | 163.0 | 59.9 | 22.5 | 8.1 | 1936 |
|  |  | Median (Population) | 1.63 |  |  |  | 11.0 | 2629 |  |  |  | 8.8 | 2103 |
|  |  | 75th percentile (More active) | 1.78 |  |  |  | 12.0 | 2868 |  |  |  | 9.7 | 2318 |
|  | 45–54 | 25th percentile (Less active) | 1.49 | 175.0 | 68.8 | 22.5 | 9.9 | 2366 | 162.0 | 59.0 | 22.5 | 8.0 | 1912 |
|  |  | Median (Population) | 1.63 |  |  |  | 10.8 | 2581 |  |  |  | 8.8 | 2103 |
|  |  | 75th percentile (More active) | 1.78 |  |  |  | 11.8 | 2820 |  |  |  | 9.6 | 2294 |
|  | 55–64 | 25th percentile (Less active) | 1.49 | 174.0 | 68.3 | 22.6 | 9.0 | 2151 | 161.0 | 58.0 | 22.4 | 7.9 | 1888 |
|  |  | Median (Population) | 1.63 |  |  |  | 9.8 | 2342 |  |  |  | 8.7 | 2079 |
|  |  | 75th percentile (More active) | 1.78 |  |  |  | 10.7 | 2557 |  |  |  | 9.5 | 2271 |
| Military of UK, 2016 | 17.8–26.4 | General population, 25th percentile | 1.49 | 178.0 | 75.7 | 23.9 | 10.9 | 2600 | 165.0 | 60.0 | 22.0 | 8.4 | 2000 |
|  |  | General population, Median | 1.63 |  |  |  | 12.0 | 2900 |  |  |  | 9.2 | 2200 |
|  |  | General population, 75th percentile | 1.78 |  |  |  | 13.1 | 3100 |  |  |  | 10.1 | 2400 |
|  |  | Active service, 25th percentile | 1.90 |  |  |  | 14.0 | 3300 |  |  |  | 10.8 | 2600 |
|  |  | Active service, Median | 2.08 |  |  |  | 15.2 | 3600 |  |  |  | 11.7 | 2800 |
|  |  | Active service, 75th percentile | 2.16 |  |  |  | 15.9 | 3800 |  |  |  | 12.2 | 2900 |
|  |  | Military training courses A, 25th percentile | 2.15 |  |  |  | 15.8 | 3800 |  |  |  | 12.1 | 2900 |
|  |  | Military training courses A, Median | 2.32 |  |  |  | 17.0 | 4100 |  |  |  | 13.1 | 3100 |
|  |  | Military training courses A, 75th percentile | 2.44 |  |  |  | 17.9 | 4300 |  |  |  | 13.8 | 3300 |
|  |  | Military training courses B, 25th percentile | 2.51 |  |  |  | 18.4 | 4400 |  |  |  | 14.2 | 3400 |
|  |  | Military training courses B, Median | 2.62 |  |  |  | 19.2 | 4600 |  |  |  | 14.8 | 3500 |
|  |  | Military training courses B, 75th percentile | 2.78 |  |  |  | 20.4 | 4900 |  |  |  | 15.7 | 3800 |
| Civilian of USA, 2002, 2005 | 16 | Sedentary-Very active | 1.25–2.20 | 174.0 | 60.9 | 20.1 | 9.7–15.3 | 2320–3663 | 163.0 | 53.9 | 20.3 | 7.2–12.1 | 1729–2883 |
|  | 17 |  |  | 175.0 | 64.6 | 21.1 | 9.9–15.7 | 2366–3754 | 163.0 | 55.1 | 20.7 | 7.2–12.0 | 1710–2871 |
|  | 18 |  |  | 176.0 | 67.2 | 21.7 | 10.0–15.9 | 2383–3804 | 163.0 | 56.2 | 21.2 | 7.1–12.0 | 1690–2858 |
|  | 30≧ |  |  | 145.0 | 38.9 | 18.5 | 7.4–10.3 | 1777–2450 | 145.0 | 38.9 | 18.5 | 6.5–9.2 | 1563–2201 |
|  |  |  |  | 145.0 | 52.5 | 25.0 | 8.3–11.6 | 1994–2771 | 145.0 | 52.5 | 25.0 | 7.1–10.0 | 1691–2386 |
|  |  |  |  | 150.0 | 41.6 | 18.5 | 7.7–10.7 | 1848–2554 | 150.0 | 41.6 | 18.5 | 6.8–9.6 | 1625–2291 |
|  |  |  |  | 150.0 | 56.2 | 25.0 | 8.7–12.1 | 2080–2898 | 150.0 | 56.2 | 25.0 | 7.4–10.4 | 1762–2489 |
|  |  |  |  | 155.0 | 44.4 | 18.5 | 8.0–11.1 | 1919–2661 | 155.0 | 44.4 | 18.5 | 7.1–10.0 | 1688–2382 |
|  |  |  |  | 155.0 | 60.0 | 25.0 | 9.1–12.7 | 2168–3028 | 155.0 | 60.0 | 25.0 | 7.7–10.8 | 1834–2593 |
|  |  |  |  | 160.0 | 47.4 | 18.5 | 8.3–11.6 | 1993–2769 | 160.0 | 47.4 | 18.5 | 7.3–10.4 | 1752–2474 |
|  |  |  |  | 160.0 | 64.0 | 25.0 | 9.4–13.2 | 2257–3160 | 160.0 | 64.0 | 25.0 | 8.0–11.3 | 1907–2699 |
|  |  |  |  | 165.0 | 50.4 | 18.5 | 8.7–12.0 | 2068–2880 | 165.0 | 50.4 | 18.5 | 7.6–10.7 | 1816–2567 |
|  |  |  |  | 165.0 | 68.0 | 25.0 | 9.8–13.8 | 2349–3296 | 165.0 | 68.0 | 25.0 | 8.3–11.7 | 1981–2807 |
|  |  |  |  | 170.0 | 53.5 | 18.5 | 9.0–12.5 | 2144–2993 | 170.0 | 53.5 | 18.5 | 7.9–11.1 | 1881–2662 |
|  |  |  |  | 170.0 | 72.2 | 25.0 | 10.2–14.4 | 2442–3434 | 170.0 | 72.2 | 25.0 | 8.6–12.2 | 2057–2916 |
|  |  |  |  | 175.0 | 56.7 | 18.5 | 9.3–13.0 | 2222–3108 | 175.0 | 56.7 | 18.5 | 8.2–11.5 | 1948–2758 |
|  |  |  |  | 175.0 | 76.5 | 25.0 | 10.6–15.0 | 2538–3576 | 175.0 | 76.5 | 25.0 | 8.9–12.7 | 2134–3028 |
|  |  |  |  | 180.0 | 59.9 | 18.5 | 9.6–13.5 | 2301–3225 | 180.0 | 59.9 | 18.5 | 8.4–11.9 | 2015–2855 |
|  |  |  |  | 180.0 | 81.0 | 25.0 | 11.0–15.6 | 2636–3720 | 180.0 | 81.0 | 25.0 | 9.3–13.1 | 2211–3140 |
|  |  |  |  | 185.0 | 63.3 | 18.5 | 10.0–14.0 | 2382–3344 | 185.0 | 63.3 | 18.5 | 8.7–12.4 | 2082–2954 |
|  |  |  |  | 185.0 | 85.5 | 25.0 | 11.4–16.2 | 2735–3867 | 185.0 | 85.5 | 25.0 | 9.6–13.6 | 2290–3255 |
| Military of USA, 1985 | 17–50 | Moderate activity | 1.80 | ― | 60–79 | ― | 13.4(11.7–15.1) | 3200  (2800–3600) | ― | 46–63 | ― | 10.0 (8.4–11.7) | 2400  (2000–2800) |
| Military of USA, 2001 | 17–50 | General / routine+ | ― | 175.0 | 79.0 | 25.8 | 13.6 | 3250 | 163.0 | 62.0 | 23.3 | 9.6 | 2300 |
|  |  | Light activity | 1.70 |  |  |  | 12.6 | 3000 |  |  |  | 9.2 | 2200 |
|  |  | Moderate activity | 1.80 |  |  |  | 13.6 | 3250 |  |  |  | 9.6 | 2300 |
|  |  | Heavy activity | 2.20 |  |  |  | 16.5 | 3950 |  |  |  | 11.3 | 2700 |
|  |  | Exceptionally-heavy activity | 2.50 |  |  |  | 19.2 | 4600 |  |  |  | 13.2 | 3150 |
| Military of USA, 2017 | 19–30 | General / routine^+^ | ― | 175.0 | 85.0 | 27.8 | 14.2 | 3400 | 163.0 | 69.0 | 26.0 | 9.6 | 2300 |
|  |  | Light activity | 1.70 |  |  |  | 12.6 | 3000 |  |  |  | 8.8 | 2100 |
|  |  | Moderate activity | 1.80 |  |  |  | 14.2 | 3400 |  |  |  | 9.6 | 2300 |
|  |  | Heavy activity | 2.20 |  |  |  | 15.5 | 3700 |  |  |  | 11.3 | 2700 |
|  |  | Exceptionally-heavy activity | 2.50 |  |  |  | 19.7 | 4700 |  |  |  | 12.6 | 3000 |
| NATO,　2010 | 19–50 | Civilian norm | 1.60 | 175.0 | 79.0 | 25.8 | 12.1 | 2900 | ― | ― | ― | ― | ― |
|  |  | Normal operations | 2.00 |  |  |  | 15.1 | 3600 | ― | ― | ― | ― | ― |
|  |  | Combat or special forces operations | 2.40 |  |  |  | 20.5 | 4900 | ― | ― | ― | ― | ― |
|  |  | Hot environment | ― |  |  |  | 20.5 | 4900 | ― | ― | ― | ― | ― |
|  |  | Cold environment | ― |  |  |  | 20.5 | 4900 | ― | ― | ― | ― | ― |
|  |  | High–altitude environment | ― |  |  |  | 19.7 | 4700 | ― | ― | ― | ― | ― |
| Target population: Healthy military personnel without any illnesses, excluding pregnant female and breastfeeding female.  1 kcal=4.184 KJ (Food and Agriculture Organization of the United Nations [FAO] / World Health Organization [WHO] Joint Special Expert Committee Report)  *The target age category was male and female between the ages of 17 and 55 years, but the majority of adults were 19-30 years old, with 17–18 years old being defined as adolescence.  **Because it is “believed to approach the limit of human endurance,” it can be applied to male personnel responsible for special military activities. Therefore, category V is set only for adult males.  ^#^Only the median value is listed.  ^+^Since there is no description of the PAL value for General/routine, the exact value is unknown, but the standard PAL for civilians[11] is Secondary (1.25), Low Active (1.50), Active (1.75) and Very Active (2.20), a range of 1.25–1.50 is possible.  ^‡^Combat or special forces operations  Abbreviation:  ANR, Average Nutrient Requirement; EAR, Equivalent to Estimated Average Requirement; EER, Equivalent to Estimated Energy Requirement Military DRV, Military Dietary Reference Value; MRDI, Military Recommended Dietary Intake; PAL, physical activity level | | | | | | | | | | | | | |

**Table S3. Reference body size and estimated formula in Dietary Reference Intakes (DRI) for military operations**

|  | Reference (Heigh, Weight, BMI) | Equation |
| --- | --- | --- |
| Australia | Australian Military Personnel  Males: 180 cm, 78.0 kg, 24.1 kg/m^2^ | Schofield's formula  〖Male 18–29 years〗BMR＝(0.063×weight[kg]+2.896)×1000/4.186  〖Male 30–59 years〗BMR＝(0.048×weight[kg]+3.653)×1000/4.186  〖Female 18–29 years〗BMR＝(0.062×weight[kg]+2.036)×1000/4.186  〖Female 30–59 years〗BMR＝(0.034×weight[kg]+3.538)×1000/4.186 |
| UK | British Military Personnel  Males: 178 cm, 75.7 kg, 23.9 kg/m^2^  Females: 165 cm, 60.0 kg, 22.0 kg/m^2^ | Henry's formula  〖Male 18–30 years〗BMR＝16.0×weight[kg]+545  〖Male 31–60 years〗BMR＝14.2×weight[kg]+593  〖Female 18–30 years〗BMR＝13.1×weight[kg]+558  〖Female 31–61 years〗BMR＝9.7×weight[kg]+694 |
| US | America's Military Personnel (2017)  Males: 175 cm, 85.0 kg, 27.8 kg/m^2^  Females: 163 cm, 69.0 kg, 26.0 kg/m^2^ | Formula in USA and Canada’s DRI  〖Male 17–18 years〗  TEE＝88.5-61.9×age(years)+PAL(26.7×weight[kg]+903×heigh [m])  〖Male 19–30 years〗  TEE＝662-9.53×age(years)+PAL(15.9×weight[kg]+540×heigh[m])  〖Female 17–18 years〗  TEE＝153.3-30.8×age(years)+PAL(10.0×weight[kg]+934×heigh[m])  〖Female 19–30 years〗  TEE＝354-6.91×age(years)+PAL(9.36×weight[kg]+726×heigh[m]) |
| NATO | America's Military Personnel (2001)  Males: 175 cm, 79.0 kg, 25.8 kg/m^2^ | Schofield's formula^*^  〖Male 30–59 years〗BMR＝(0.048×weight[kg]+3.653)×1000/4.186 |
| ^*^The basal metabolic standard values for personnel engaged in combat or special forces operations are not based on Schofield's equation, but are based on research reports using the double-labeled water method for personnel engaged in combat operations. | | |

**Table S4. Protein** **in Dietary Reference Intakes (DRI) for civilians and military (/day)**

| DRI | Age  (years) | Category (PAL) | Males | | | | | Females | | | | |
| --- | --- | --- | --- | --- | --- | --- | --- | --- | --- | --- | --- | --- |
|  |  |  | EAR | | RDA | | AMDR^※^ | EAR | | RDA | | AMDR^※^ |
|  |  |  | g | g/kg | g | g/kg | %E | g | g/kg | g | g/kg | %E |
| Civilian of Australia, 2006 | 14–18 | ― | 49 | 0.76 | 65 | 0.99 | 15–25^*^ | 35 | 0.62 | 45 | 0.77 | 15–25^*^ |
|  | 19–70 | ― | 52 | 0.68 | 64 | 0.84 |  | 37 | 0.60 | 46 | 0.75 |  |
| Military of Australia, 2009 | 17–18 | Low physical activity (1.5) | ― | ― | 110–147 | ― | 15–20 | ― | ― | 79–106 | ― | 15–20 |
|  |  | Moderately active (1.8) | ― | ― | 119–162 | ― | 14–19 | ― | ― | 86–117 | ― | 14–19 |
|  |  | Very active (2.1) | ― | ― | 126–175 | ― | 13–18 | ― | ― | 92–127 | ― | 13–18 |
|  |  | Extreme activity (2.4) | ― | ― | 138–195 | ― | 12–17 | ― | ― | 95–135 | ― | 12–17 |
|  | 19–30 | Low physical activity (1.5) | ― | ― | 106–141 | ― | 15–20 | ― | ― | 75–100 | ― | 15–20 |
|  |  | Moderately active (1.8) | ― | ― | 115–156 | ― | 14–19 | ― | ― | 82–112 | ― | 14–19 |
|  |  | Very active (2.1) | ― | ― | 122–169 | ― | 13–18 | ― | ― | 88–122 | ― | 13–18 |
|  |  | Extreme activity (2.4) | ― | ― | 134–190 | ― | 12–17 | ― | ― | 92–130 | ― | 12–17 |
|  |  | Believed to approach the limit of human endurance (3.2)^**^ | ― | ― | 162–235 | ― | 11–16 | ― | ― | ― | ― | ― |
|  | 17–30 | High-altitude environment^***^ | ― | ― | ― | ― | 15 | ― | ― | ― | ― | 15 |
| Civilian of UK, 1991^#^ | 15–18 | ― | 46.1 | ― | 55.2 | ― | (15) | 37.1 | ― | 45.4 | ― | (15) |
|  | 19–50 | ― | 44.4 |  | 55.5 | ― | (15) | 36.0 | ― | 45.0 | ― | (15) |
|  | 50< | ― | 42.6 |  | 53.3 | ― | (15) | 37.2 | ― | 46.5 | ― | (15) |
| Military of UK, 2016, 2020 | 16–60 | General population (1.6) | ― | ― | ― | ― | 15 | ― | ― | ― | ― | 15 |
|  |  | Active service (2.1) | ― | ― | ― | ― | 13.5–15 | ― | ― | ― | ― | 13.5–15 |
|  |  | Military training courses A (2.3) | ― | ― | ― | ― | 12–15 | ― | ― | ― | ― | 12–15 |
|  |  | Military training courses B (2.6) | ― | ― | ― | ― | 10–15 | ― | ― | ― |  | 10–15 |
| Civilian of USA, 2002, 2005^+^ | 14–18 | ― | ― | 0.73 | 52 | 0.85 | 10–35 | ― | ― | 46 | 0.85 | 10–35 |
|  | 19–70 | ― | ― | 0.66 | 56 | 0.80 | 10–35 | ― | 0.66 | 46 | 0.80 | 10–35 |
| Military of USA, 1985^+^ | 17–50 | ― | ― | ― | 100 | 0.80 | ― | ― | ― | 80 | 0.80 | ― |
| Military of USA, 2001^+^ | 17–50 | ― | ― | ― | 91 (63–119) | ― | 10–15 | ― | ― | 72 (50–93) | ― | 10–15 |
| Military of USA, 2017^+^ | 19–30 | ― | ― | ― | 102 (68–136) | 0.80–1.60 | 10–35 | ― | ― | 83 (55–110) | 0.80–1.60 | 10–35 |
| NATO, 2010^‡^ | 19–50 | Civilian norm (1.6) | ― | ― | 144 (108–179) | ― | 15–25 | ― | ― | ― | ― | ― |
|  |  | Normal operations (2.0) | ― | ― | 180 (135–225) | ― |  | ― | ― | ― | ― | ― |
|  |  | Combat or special forces operations (2.4) | ― | ― | 246 (184–307) | ― |  | ― | ― | ― | ― | ― |
|  |  | Hot environment | ― | ― | 246 (184–307) | ― |  | ― | ― | ― | ― | ― |
|  |  | Cold environment | ― | ― | 246 (184–307) | ― |  | ― | ― | ― | ― | ― |
|  |  | High-altitude environment | ― | ― | 178 (178–296) | ― | 15 |  |  | ― |  |  |
| The targets were healthy adults, excluding pregnant women and lactating women, but the criteria for the age range was organized to include 16 years old and over and under 65 years old, including 16 years old, which was the minimum age in the military report.  ^※^In Civilian of UK, it is set as "population average". ^*^Target age categories for AMDR have not been established.  ^**^“Believed to approach the limit of human endurance (3.2)” is limited to male adults (19–30 years).  ^***^15% energy/day was recommended for military operations at altitudes above 3000 m.  ^‡^Targeted only at male.  ^+^It is formulated in PAL category “Moderate (1.8)”.  ^#^Although the population average (% energy) of protein has not been established, the energy ratio of fat and carbohydrate has been established, so the energy ratio of protein is inevitably 15% (SACN, 1991).  Abbreviation:  AMDR, Acceptable Macronutrient Distribution Range; EAR, Estimated Average Requirement; RDA，Recommended Dietary Allowance | | | | | | | | | | | | |

**Table S5. Fat in Dietary Reference Intakes (DRI) for civilians and military (/day)**

| DRI | Term | Age  (years) | Category (PAL) | Males | | Females | |
| --- | --- | --- | --- | --- | --- | --- | --- |
|  |  |  |  | %E | g | %E | g |
| Civilian of Australia, 2006 | AMDR | ―^*^ | ― | 20–35 | ― | 20–35 | ― |
| Military of Australia, 2006 | MRDI | 17–18 | Low physical activity (1.5) | 25–35 | ― | 25–35 | ― |
|  |  |  | Moderately active (1.8) | 24–34 | ― | 24–34 | ― |
|  |  |  | Very active (2.1) | 23–33 | ― | 23–33 | ― |
|  |  |  | Extreme activity (2.4) | 22–32 | ― | 22–32 | ― |
|  |  | 19–30 | Low physical activity (1.5) | 25–35 | ― | 25–35 | ― |
|  |  |  | Moderately active (1.8) | 24–34 | ― | 24–34 | ― |
|  |  |  | Very active (2.1) | 23–33 | ― | 23–33 | ― |
|  |  |  | Extreme activity (2.4) | 22–32 | ― | 22–32 | ― |
|  |  |  | Believed to approach the limit of human endurance (3.2)^**^ | 21–31 | ― | ― | ― |
|  |  | 17–30 | High–altitude environment ^**^ | 20 | ― | 20 | ― |
| Civilian of UK, 1991^#^ (without alcohol) | Population average | Adults | ― | 33 (35) | ― | 33 (35) | ― |
| Military of UK, 2016, 2020 | Military DRV | 16–60 | General population (1.6) | 35 | ― | 35 | ― |
|  |  |  | Active service (2.1) | 31.5–35 | ― | 31.5–35 | ― |
|  |  |  | Military training courses A (2.3) | 28–35 | ― | 28–35 | ― |
|  |  |  | Military training courses B (2.6) | 25–35 | ― | 25–35 | ― |
| Civilian of USA, 2002, 2005^+^ | AMDR | 14–70 | ― | 10–35 | ― | 10–35 | ― |
| Military of USA, 1985^+^ | MRDA | 17–50 | ― | <35 | ― | <35 | ― |
| Military of USA, 2001^+^ | MDRI | 17–50 | ― | <30 | ― | <30 | ― |
| Military of USA, 2017^+^ | MDRI | 19–30 | ― | <30 | <113 (100–157) | <30 | <77 (70–100) |
| NATO, 2010^‡^ | AMDR | 19–50 | Civilian norm (1.6) | 20–35 | 88 (64–111) | ― | ― |
|  |  |  | Normal operations (2.0) | 20–35 | 110 (80–140) | ― | ― |
|  |  |  | Combat or special forces operations (2.4) | 20–35 | 150 (109–191) | ― | ― |
|  |  |  | Hot environment | 20–35 | 150 (109–191) | ― | ― |
|  |  |  | Cold environment | 20–35 | 150 (109–191) | ― | ― |
|  |  |  | High–altitude environment | ≤25 | 132 (105–184) | ― | ― |
| The targets were healthy adults, excluding pregnant women and lactating women, but the criteria for the age range was organized to include 16 years old and over and under 65 years old, including 16 years old, which was the minimum age in the military report.  ^*^Target age categories for AMDR have not been established.  ^**^“Believed to approach the limit of human endurance (3.2)” is limited to male adults (19–30 years).  ^#^No target age category has been established, but since adults are the target population and alcohol intake varies among individuals, alcohol-free criteria have also been established, and this is considered the general population standard in the DRI for military operations.  ^+^It is formulated in PAL category “Moderate (1.8)”.  ^++^MDRI has not been formulated.  ^‡^Targeted only at male.  Abbreviation:  AMDR, Acceptable Macronutrient Distribution Range; MDRI, Military Dietary Reference Intake; Military DRV, Military Dietary Reference Value; MRDA, Military Recommended Dietary Allowances | | | | | | | |

**Table S6. Fatty acids for which standard values have been established in Dietary Reference Intakes (DRI) for civilians and military (/day)**

| DRI | Age (years) | Category (PAL) | Males | | | | | | | | | | Females | | | | | |
| --- | --- | --- | --- | --- | --- | --- | --- | --- | --- | --- | --- | --- | --- | --- | --- | --- | --- | --- |
|  |  |  | Saturated Fat | | PUFA^*^ | | Total LC-  n-3^**^ | | Linoleic acid | | α-linolenic acid | | Total LC-n-3^**^ | | Linoleic acid | | α-linolenic acid | |
|  |  |  | term | g | term | g | term | mg | term | g | term | g | term | mg | term | g | term | g |
| Civilian of Australia, 2006 | 14–18 | ― | ― | ― | ― | ― | AI | 70 | AI | 12 | AI | 1 | AI | 85 | AI | 8 | AI | 1 |
|  | 19–70 | ― | ― | ― | ― | ― |  | 160 |  | 13 |  | 1 |  | 90 |  | 8 |  | 1 |
|  | ― | ― | ― | ― | ― | ― | UL | 3000 | ― | ― | ― | ― | UL | 3000 | ― | ― | ― | ― |
|  | ― | ― | ― | ― | ― | ― | SDT | 610 | ― | ― | ― | ― | SDT | 430 | ― | ― | ― | ― |
| Civilian of USA, 2002, 2005^+^ | 14–18 | ― | ― | ― | ― | ― | ― | ― | AI | 16 | AI | 2 | ― | ― | AI | 11 | AI | 1 |
|  | 19–30 | ― | ― | ― | ― | ― | ― | ― |  | 17 |  | 2 | ― | ― |  | 12 |  | 1 |
|  | 31–50 | ― | ― | ― | ― | ― | ― | ― |  | 17 |  | 2 | ― | ― |  | 12 |  | 1 |
|  | 51–70 | ― | ― | ― | ― | ― | ― | ― |  | 14 |  | 2 | ― | ― |  | 11 |  | 1 |
| Military of USA, 2017^+^ | 19–30 | ― | ― | ― | ― | ― | ― | ― | MDRI | 17 | MDRI | 2 | ― | ― | MDRI | 12 | MDRI | 1 |
|  | 19–30 | ― | ― | ― | ― | ― | ― | ― | NSOR | 17 | NSOR | 2 | ― | ― | NSOR | 17 | NSOR | 2 |
|  | 19–30 | ― | ― | ― | ― | ― | ― | ― | NSRR | 9 | NSRR | 1 | ― | ― | NSRR | 9 | NSRR | 1 |
| NATO, 2010^‡^ | 19–50 | Civilian norm (1.6) | AI | <32 | AI | 22  (13–32) | AI | ## | AI | 13 | AI | 1 | ― | ― | ― | ― | ― | ― |
|  |  | Normal operations (2.0) |  | <40 |  | 28  (16–40) |  | ## |  | 13 |  | 1 | ― | ― | ― | ― | ― | ― |
|  |  | Combat or special forces operations (2.4) |  | <55 |  | 38  (22–54) |  | ## |  | 13 |  | 1 | ― | ― | ― | ― | ― | ― |
|  |  | Hot environment |  | <55 |  | 38  (22–54) |  | ## |  | 13 |  | 1 | ― | ― | ― | ― | ― | ― |
|  |  | Cold environment |  | <55 |  | 38  (22–54) |  | ## |  | 13 |  | 1 | ― | ― | ― | ― | ― | ― |
|  |  | High-altitude environment |  | <52 |  | 37  (21–53) |  | ## |  | 13 |  | 1 | ― | ― | ― | ― | ― | ― |
| The targets were healthy adults, excluding pregnant women and lactating women, but the criteria for the age range was organized to include 16 years old and over and under 65 years old, including 16 years old, which was the minimum age in the military report.  ^+^It is formulated in PAL category “Moderate (1.8)”.  ^++^Target age categories for AMDR have not been established.  ^‡^Targeted only at male.  ^*^PUFA (polyunsaturated fatty acid)  ^**^Total LC-n-3 (DHA+EPA+DPA) | | | | | | | | | | | | | | | | | | |

**Table S7. Carbohydrate** **in Dietary Reference Intakes (DRI) for civilians and military (/day)**

| DRI | Term | Age  (years) | Category (PAL) | Males | | Females | |
| --- | --- | --- | --- | --- | --- | --- | --- |
|  |  |  |  | %E | g | %E | g |
| Civilian of Australia, 2006 | AMDR | ―^*^ | ― | 45–65 | ― | 45–65 | ― |
| Military of Australia, 2006 | MRDI | 17–18 | Low physical activity (1.5) | 50–55 | 266–292 | 50–55 | 281–309 |
|  |  |  | Moderately active (1.8) | 52–57 | 325–356 | 52–57 | 341–374 |
|  |  |  | Very active (2.1) | 54–59 | 388–424 | 54–59 | 405–443 |
|  |  |  | Extreme activity (2.4) | 56–61 | 455–496 | 56–61 | 473–515 |
|  |  | 19–30 | Low physical activity (1.5) | 50–55 | 375–413 | 50–55 | 391–430 |
|  |  |  | Moderately active (1.8) | 52–57 | 455–499 | 52–57 | 471–517 |
|  |  |  | Very active (2.1) | 54–59 | 540–590 | 54–59 | 557–608 |
|  |  |  | Extreme activity (2.4) | 56–61 | 665–724 | 56–61 | 683–743 |
|  |  |  | Believed to approach the limit of human endurance (3.2)^**^ | 58–63 | 906–984 | ― | ― |
|  |  | 17–30 | High–altitude environment ^**^ | 65 | ― | 65 | ― |
| Civilian of UK, 2015^#^ (without alcohol) | Population average | Adults | ― | 47 (50) | 309 (329) | 47 (50) | 252 (268) |
| Civilian of USA, 2016, 2020 | Military DRV | 16–60 | General population (1.6) | 50 | ― | 50 | ― |
|  |  |  | Active service (2.1) | 50–55 | ― | 50–55 | ― |
|  |  |  | Military training courses A (2.3) | 50–60 | ― | 50–60 | ― |
|  |  |  | Military training courses B (2.6) | 50–65 | ― | 50–65 | ― |
| Civilian of UK, 2002, 2005^+^ | EAR | 14–70 | ― | ― | 100 | ― | 100 |
|  | RDA | 14–70 | ― | ― | 130 | ― | 130 |
|  | AMDR | 14–70 | ― | 45–65 | ― | 45–65 | ― |
| Civilian of USA, 1985^+^ | MRDA | 17–50 | ― | 50–55 | ― | 50–55 | ― |
| Military of USA, 2017^+^ | MDRI | 19–30 | ― | 50–55 | 510 (340–680) | 50–55 | 414 (276–552) |
| NATO, 2010^‡^ | AMDR | 19–50 | Civilian norm (1.6) | 45–65 | 395 (323–466) | ― | ― |
|  |  |  | Normal operations (2.0) | 45–65 | 494 (404–584) | ― | ― |
|  |  |  | Combat or special forces operations (2.4) | 45–65 | 675 (552–797) | ― | ― |
|  |  |  | Hot environment | 45–65 | 675 (552–797) | ― | ― |
|  |  |  | Cold environment | 45–65 | 675 (552–797) | ― | ― |
|  |  |  | High–altitude environment | 60 | 711 (533–770) | ― | ― |
| The targets were healthy adults, excluding pregnant women and lactating women, but the criteria for the age range was organized to include 16 years old and over and under 65 years old, including 16 years old, which was the minimum age in the military report.  ^*^Target age categories for AMDR have not been established.  ^**^“Believed to approach the limit of human endurance (3.2)” is limited to male adults (19–30 years).  ^#^No target age category has been established, but since adults are the target population and alcohol intake varies among individuals, alcohol-free criteria have also been established, and this is considered the general population standard in the DRI for military operations.  ^+^It is formulated in PAL category “Moderate (1.8)”.  ^++^MDRI has not been formulated in 2001.  ^‡^Targeted only at male.  Abbreviation:  AMDR, Acceptable Macronutrient Distribution Range; EAR, Estimated Average Requirement; MDRI, Military Dietary Reference Intake; Military DRV, Military Dietary Reference Value; MRDA, Military Recommended Dietary Allowances; NSOR, Nutritional Standards for Operational Ration; NSRR, Nutritional Standards for Restricted Ration; RDA, Recommended Dietary Allowance | | | | | | | |

**Table S8.** **Macronutrient energy distribution in Dietary reference intakes (DRI) for civilians and military (% Energy/day)**

| DRI | Term | Age (years) | Category (PAL) | Protein | Fat | Carbohydrate |
| --- | --- | --- | --- | --- | --- | --- |
| Civilian of Australia, 2006^*^ | AMDR | － | － | 15–25 | 20–35 | 45–65 |
| Military of Australia, 2009 | MRDI | 17–30 | Low physical activity (1.5) | 15–20 | 25–35 | 50–55 |
|  |  |  | Moderately active (1.8) | 14–19 | 24–34 | 52–57 |
|  |  |  | Very active (2.1) | 13–18 | 23–33 | 54–59 |
|  |  |  | Extreme activity (2.4) | 12–17 | 22–32 | 56–61 |
|  |  |  | Believed to approach the limit of human endurance (3.2)^**^ | 11–16 | 21–31 | 58–63 |
|  |  |  | High–altitude environment^***^ | 15 | 20 | 65 |
| Civilian of UK, 1991^#^ (without alcohol) | Population average | － | － | － | 33 (35) | 50 (50) |
| Military of UK, 2016, 2020 | Military DRV | 16–60 | General population (1.6) | 15 | 35 | 50 |
|  |  |  | Active service (2.1) | 13.5–15 | 31.5–35 | 50–55 |
|  |  |  | Military training courses A (2.3) | 12–15 | 28–35 | 50–60 |
|  |  |  | Military training courses B (2.6) | 10–15 | 25–35 | 50-65 |
| Civilian of USA, 2002, 2005 | AMDR | 14–70 | － | 10–35 | 20–35 | 45–65 |
| Military of USA, 1985^+^ | MRDA | 17–50 | － | － | <35 | 50–55 |
| Military of USA, 2001^+^ | MDRI | 17–50 | － | 10–15 | <35 | ≧55 |
| Military of USA, 2017^+^ | MDRI | 19–30 | － | 10–35 | <30 | 50–55 |
| NATO, 2010^‡^ | AMDR | 19–50 | － | 15–25 | 20–35 | 45–65 |
|  |  |  | High–altitude environment^‡‡^ | 15 | ≤25 | 60 |
| The targets were healthy adults, excluding pregnant women and lactating women, but the criteria for the age range was organized to include 16 years old and over and under 65 years old, including 16 years old, which was the minimum age in the military report.  ^*^Target age category was not set.  ^**^“Believed to approach the limit of human endurance (3.2)” is limited to male adults (19–30 years).  ^***^15:20:65 is recommended for military operations at altitudes above 3000 m.  ^#^The target age category has not been established, but adults are covered, and the DRI for civilians has a standard that includes alcohol (F 33%, C 47%) and a standard that does not (F 35%, C 50%) because of individual differences in alcohol consumption, and in the military the 'not included' standard is the standard for the general population.  ^+^It is formulated in PAL category “Moderate (1.8)”.  ^‡^Targeted only at male.  ^‡‡^When engaging in military activities in high-altitude environments, a ratio of 15:≦25:60 is recommended.  Abbreviation:  AMDR, Acceptable Macronutrient Distribution Range; MRDA, Military Recommended Dietary Allowances; Military Recommended Dietary Intake | | | | | | |

**Table S9. Dietary fiber in Dietary Reference Intakes (DRI) for civilians and military (g/day)**

| DRI | Age (years) | Males | | | Females | | |
| --- | --- | --- | --- | --- | --- | --- | --- |
|  |  | AI | Other | | AI | Other | |
| Civilian of Australia, 2006 | 14–18 | 28 | SDT^*^ | 38 | 22 | SDT^*^ | 28 |
|  | 19–70 | 30 |  |  | 25 |  |  |
| Military of Australia, 2009 | 17–18 | 30 | － | － | 25 | － | － |
|  | 19–30 | 30 | － | － | 25 | － | － |
| Civilian of UK, 2015^#^ | 15–16 | － | DRV | 25 | － | DRV | 25 |
|  | 17–60 | － |  | 30 | － |  | 30 |
| Civilian of USA, 2002, 2005 | 14–18 | 38 | － | － | 26 | － | － |
|  | 19–50 | 30 | － | － | 25 | － | － |
|  | 51–70 |  | － | － | 21 | － | － |
| Military of USA, 2017^++^ | 19–30 | 34 | － | － | 28 | － | － |
| NATO, 2010^‡^ | 19–50 | 30 | － | － | － | － | － |
| The targets were healthy adults, excluding pregnant women and lactating women, but the criteria for the age range was organized to include 16 years old and over and under 65 years old, including 16 years old, which was the minimum age in the military report.  ^*^Target age category was not set.  ^#^No formulation of DRI for military operations has been made. ^++^Dietary fiber was not included in the DRI before the revision (1985, 2001).  ^‡^Targeted only at male.  Abbreviation:  AI, Adequate Intake; DRV, Dietary Reference Value; SDT, Suggested Dietary Target; NSOR, Nutritional Standards for Operational Ration; NSRR, Nutritional Standards for Restricted Ration | | | | | | | |

**Table S10. Vitamin B_1_ in Dietary Reference Intakes (DRI) for civilians and military (mg/day)**

| DRI | Age (years) | Category (PAL) | Males | | | | Females | | | |
| --- | --- | --- | --- | --- | --- | --- | --- | --- | --- | --- |
|  |  |  | EAR | RDA | Other | | EAR | RDA | Other | |
| Civilian of Australia, 2006 | 14–70 | － | 1.0 | 1.2 | － | － | 0.9 | 1.1 | － | － |
| Military of Australia, 2009 | 17–18 | Low physical activity (1.5) | － | 1.3 | － | － | － | 0.9 | － | － |
|  |  | Moderately active (1.8) | － | 1.5 | － | － | － | 1.1 | － | － |
|  |  | Very active (2.1) | － | 1.7 | － | － | － | 1.2 | － | － |
|  |  | Extreme activity (2.4) | － | 2.0 | － | － | － | 1.4 | － | － |
|  | 19–30 | Low physical activity (1.5) | － | 1.2 | － | － | － | 0.9 | － | － |
|  |  | Moderately active (1.8) | － | 1.4 | － | － | － | 1.0 | － | － |
|  |  | Very active (2.1) | － | 1.6 | － | － | － | 1.2 | － | － |
|  |  | Extreme activity (2.4) | － | 1.9 | － | － | － | 1.3 | － | － |
|  |  | Believed to approach the limit of human endurance (3.2)^**^ | － | 2.5 | － | － | － | － | － | － |
| Civilian of UK, 1991 (mg/1000 kcal/day)^#^ | 15–50 | － | (0.3) | 1.1  (0.4) | LRNI | (0.23) | (0.3) | 0.8  (0.4) | LRNI | (0.23) |
|  | 50< | － |  | 0.9  (0.4) |  |  |  |  |  |  |
| Civilian of USA, 1998 | 14–70 | － | 1.0 | 1.2 | － | － | 0.9 | 1.0 | － | － |
| Military of USA, 1985 | 17–50 | － | － | 1.6 | － | － | － | 1.2 | － | － |
| Military of USA, 2001 | 17–50 | － | － | 1.2 | － | － | － | 1.1 | － | － |
| Military of USA, 2017 | 19–30 | － | － | 1.2 | － | － | － | 1.1 | － | － |
| NATO, 2010 | 19–50 | － | － | 1.2 | － | － | － | － | － | － |
| The targets were healthy adults, excluding pregnant women and lactating women, but the criteria for the age range was organized to include 16 years old and over and under 65 years old, including 16 years old, which was the minimum age in the military report.  ^*^“Believed to approach the limit of human endurance (3.2)” is limited to male adults (19–30 years).  ^#^No formulation of DRI for military operations has been made.    Abbreviation:  EAR, Estimated Average Requirement; LRNI, Lower Reference Nutrient Intake; NSOR, Nutritional Standards for Operational Ration; NSRR, Nutritional Standards for Restricted Ration; RDA, Recommended Dietary Allowance | | | | | | | | | | |

**Table S11. Vitamin B_2_ in Dietary Reference Intakes (DRI) for civilians and military (mg/day)**

| DRI | Age (years) | Category (PAL) | Males | | | | Females | | | |
| --- | --- | --- | --- | --- | --- | --- | --- | --- | --- | --- |
|  |  |  | EAR | RDA | Other | | EAR | RDA | Other | |
| Civilian of Australia, 2006 | 14–70 | － | 1.1 | 1.3 | － | － | 0.9 | 1.1 | － | － |
| Military of Australia, 2009 | 17–18 | Low physical activity (1.5) | － | 1.9 | － | － | － | 1.4 | － | － |
|  |  | Moderately active (1.8) | － | 2.2 | － | － | － | 1.6 | － | － |
|  |  | Very active (2.1) | － | 2.5 | － | － | － | 1.8 | － | － |
|  |  | Extreme activity (2.4) | － | 2.9 | － | － | － | 2.0 | － | － |
|  | 19–30 | Low physical activity (1.5) | － | 1.8 | － | － | － | 1.3 | － | － |
|  |  | Moderately active (1.8) | － | 2.1 | － | － | － | 1.5 | － | － |
|  |  | Very active (2.1) | － | 2.4 | － | － | － | 1.7 | － | － |
|  |  | Extreme activity (2.4) | － | 2.9 | － | － | － | 2.0 | － | － |
|  |  | Believed to approach the limit of human endurance (3.2)^**^ | － | 3.8 | － | － | － | － | － | － |
| Civilian of UK, 1991^#^ | 15–50 | － | 1.0 | 1.3 | LRNI | 0.8 | 0.9 | 1.1 | LRNI | 0.8 |
|  | 50< | － | 1.0 | 1.3 |  | 0.8 | 0.9 | 1.1 |  | 0.8 |
| Civilian of USA, 1998 | 14–18 | － | 1.1 | 1.3 | － | － | 0.9 | 1.0 | － | － |
|  | 19–70 | － |  |  | － | － |  | 1.1 | － | － |
| Military of USA, 1985 | 17–50 | － | － | 1.9 | － | － | － | 1.4 | － | － |
| Military of USA, 2001 | 17–50 | － | － | 1.3 | － | － | － | 1.1 | － | － |
| Military of USA, 2017 | 19–30 | － | － | 1.3 | － | － | － | 1.1 | － | － |
| NATO, 2010 | 19–50 | Civilian norm (1.6) | － | 1.3 | － | － | － | － | － | － |
|  |  | Normal operations (2.0) | － | 1.3 | － | － | － | － | － | － |
|  |  | Combat or special forces operations (2.4) | － | 2.5 | － | － | － | － | － | － |
|  |  | Hot environment | － | 2.5 | － | － | － | － | － | － |
|  |  | Cold environment | － | 2.5 | － | － | － | － | － | － |
|  |  | High–altitude environment | － | 2.5 | － | － | － | － | － | － |
| The targets were healthy adults, excluding pregnant women and lactating women, but the criteria for the age range was organized to include 16 years old and over and under 65 years old, including 16 years old, which was the minimum age in the military report.  ^*^“Believed to approach the limit of human endurance (3.2)” is limited to male adults (19–30 years).  ^#^No formulation of DRI for military operations has been made.  Abbreviation:  EAR, Estimated Average Requirement; LRNI, Lower Reference Nutrient Intake; RDA, Recommended Dietary Allowance | | | | | | | | | | |

**Table S12. Niacin in Dietary Reference Intakes (DRI) for civilians and military (mg/day)**

| DRI | Age (years) | Category (PAL) | Males | | | | | Females | | | | |
| --- | --- | --- | --- | --- | --- | --- | --- | --- | --- | --- | --- | --- |
|  |  |  | EAR | RDA | UL | Other | | EAR | RDA | UL | Other | |
| Civilian of Australia, 2006  (For intake from fortified foods or supplements) | 14–18 | － | 12 | 16 | 750  (30) | － | － | 11 | 14 | 750  (30) | － | － |
|  | 19–70 | － |  |  | 900  (35) | － | － |  |  | 900  (35) | － | － |
| Military of Australia, 2009 | 17–18 | Low physical activity (1.5) | － | 20 | － | － | － | － | 14 | － | － | － |
|  |  | Moderately active (1.8) | － | 23 | － | － | － | － | 17 | － | － | － |
|  |  | Very active (2.1) | － | 26 | － | － | － | － | 19 | － | － | － |
|  |  | Extreme activity (2.4) | － | 31 | － | － | － | － | 22 | － | － | － |
|  | 19–30 | Low physical activity (1.5) | － | 19 | － | － | － | － | 14 | － | － | － |
|  |  | Moderately active (1.8) | － | 22 | － | － | － | － | 16 | － | － | － |
|  |  | Very active (2.1) | － | 26 | － | － | － | － | 18 | － | － | － |
|  |  | Extreme activity (2.4) | － | 30 | － | － | － | － | 21 | － | － | － |
|  |  | Believed to approach the limit of human endurance (3.2)^**^ | － | 40 | － | － | － | － | － | － | － | － |
| Civilian of UK, 1991 (mg/1000 kcal/day)^#^ | 15–18 | － | (5.5) | 18  (6.6) | － | LRNI | (4.4) | (5.5) | 14  (6.6) | － | LRNI | (4.4) |
|  | 19–50 | － |  | 17  (6.6) | － |  |  |  | 13  (6.6) | － |  |  |
|  | 50< | － |  | 16  (6.6) | － |  |  |  | 12  (6.6) | － |  |  |
| Civilian of USA, 1998 | 14–18 | － | 12 | 16 | 30 | － | － | 11 | 14 | 30 | － | － |
|  | 19–30 | － | 12 | 16 | 35 | － | － | 11 | 14 | 35 | － | － |
|  | 31–50 | － | 12 | 16 | 35 | － | － | 11 | 14 | 35 | － | － |
|  | 51–70 | － | 12 | 16 | 35 | － | － | 11 | 14 | 35 | － | － |
| Military of USA, 1985 | 17–50 | － | － | 21 | － | － | － | － | 16 | － | － | － |
| Military of USA, 2001 | 17–50 | － | － | 16 | － | － | － | － | 14 | － | － | － |
| Military of USA, 2017 | 19–30 | － | － | 16 | － | － | － | － | 14 | － | － | － |
| NATO, 2010 | 19–50 | － | － | 16 | － | － | － | － | － | － | － | － |
| The targets were healthy adults, excluding pregnant women and lactating women, but the criteria for the age range was organized to include 16 years old and over and under 65 years old, including 16 years old, which was the minimum age in the military report.  ^*^“Believed to approach the limit of human endurance (3.2)” is limited to male adults (19–30 years).  ^#^No formulation of DRI for military operations has been made.  Abbreviation:  EAR, Estimated Average Requirement; LRNI, Lower Reference Nutrient Intake; RDA, Recommended Dietary Allowance | | | | | | | | | | | | |

**Table S13. Vitamin B_6_ in Dietary Reference Intakes (DRI) for civilians and military (mg/day)**

| DRI | Age (years) | Category (PAL) | Males | | | | | Females | | | | |
| --- | --- | --- | --- | --- | --- | --- | --- | --- | --- | --- | --- | --- |
|  |  |  | EAR | RDA | UL | Other | | EAR | RDA | UL | Other | |
| Civilian of Australia, 2006 | 14–18 | － | 1.1 | 1.3 | 40 | － | － | 1.0 | 1.2 | 40 | － | － |
|  | 19–50 | － |  |  | 50 | － | － | 1.1 | 1.3 | 50 | － | － |
|  |  | － |  |  |  | － | － |  |  |  | － | － |
|  | 51–70 | － | 1.4 | 1.7 |  | － | － | 1.3 | 1.5 |  | － | － |
| Military of Australia, 2009^*^ | 17–18 | Low physical activity (1.5) | － | 2.2 | － | － | － | － | 1.6 | － | － | － |
|  |  | Moderately active (1.8) | － | 2.4 | － | － | － | － | 1.7 | － | － | － |
|  |  | Very active (2.1) | － | 2.5 | － | － | － | － | 1.8 | － | － | － |
|  |  | Extreme activity (2.4) | － | 2.8 | － | － | － | － | 1.9 | － | － | － |
|  | 19–30 | Low physical activity (1.5) | － | 2.1 | － | － | － | － | 1.5 | － | － | － |
|  |  | Moderately active (1.8) | － | 2.3 | － | － | － | － | 1.6 | － | － | － |
|  |  | Very active (2.1) | － | 2.4 | － | － | － | － | 1.8 | － | － | － |
|  |  | Extreme activity (2.4) | － | 2.7 | － | － | － | － | 1.8 | － | － | － |
|  |  | Believed to approach the limit of human endurance (3.2)^**^ | － | 3.2 | － | － | － | － | － | － | － | － |
| Civilian of UK, 1991 (µg/g protein/day)^#^ | 15–18 | － | (13) | 1.5 (15) | － | LRNI | (11) | (13) | 1.2 (15) | － | LRNI | (11) |
|  | 19–50 | － |  | 1.4 (15) | － |  |  |  |  | － |  |  |
|  | 50< | － |  |  | － |  |  |  |  | － |  |  |
| Civilian of USA, 1998 | 14–18 | － | 1.1 | 1.3 | 80 | － | － | 1.0 | 1.2 | 80 | － | － |
|  | 19–50 | － |  |  | 100 | － | － | 1.1 | 1.3 | 100 | － | － |
|  |  | － |  |  |  | － | － |  |  |  | － | － |
|  | 51–70 | － | 1.4 | 1.7 |  | － | － | 1.3 | 1.5 |  | － | － |
| Military of USA, 1985 | 17–50 | － | － | 2.2 | － | － | － | － | 2.0 | － | － | － |
| Military of USA, 2001 | 17–50 | － | － | 1.3 | － | － | － | － | 1.3 | － | － | － |
| Military of USA, 2017 | 19–30 | － | － | 1.3 | － | － | － | － | 1.3 | － | － | － |
| NATO, 2010 | 19–50 | Civilian norm (1.6) | － | 1.3 | － | － | － | － | － | － | － | － |
|  |  | Normal operations (2.0) | － | 1.3 | － | － | － | － | － | － | － | － |
|  |  | Combat or special forces operations (2.4) | － | 2.6 | － | － | － | － | － | － | － | － |
|  |  | Hot environment |  |  |  |  |  |  |  |  |  |  |
|  |  | Cold environment |  |  |  |  |  |  |  |  |  |  |
|  |  | High–altitude environment |  |  |  |  |  |  |  |  |  |  |
| The targets were healthy adults, excluding pregnant women and lactating women, but the criteria for the age range was organized to include 16 years old and over and under 65 years old, including 16 years old, which was the minimum age in the military report.  ^*^Vitamin B_6_ in Australia's MDRI was calculated based on a calculation of 0.02 mg/g of protein.  ^**^“Believed to approach the limit of human endurance (3.2)” is limited to male adults (19–30 years).  ^#^No formulation of DRI for military operations has been made.    Abbreviation:  EAR, Estimated Average Requirement; LRNI, Lower Reference Nutrient Intake; RDA, Recommended Dietary Allowance | | | | | | | | | | | | |

**Table S14. Sodium in Dietary Reference Intakes (DRI) for civilians and military (mg/day)**

| DRI | Term | Age (years) | Category (PAL) | Males | | Females | |
| --- | --- | --- | --- | --- | --- | --- | --- |
|  |  |  |  | Sodium (mg) | Salt equivalent(g) | Sodium (mg) | Salt equivalent(g) |
| Civilian of Australia, 2006, 2017 * | AI | 14–18 | ― | 460–920 | 1.1–2.3 | 460–920 | 1.1–2.3 |
|  |  | Adults | ― | 460–920 | 1.1–2.3 | 460–920 | 1.1–2.3 |
|  | SDT | Adults | ― | 2000 | 4.9 | 2000 | 4.9 |
|  | UL | 14–18 | ― | 2300 | 5.6 | 2300 | 5.6 |
|  |  | 19–70 | ― | ― | ― | ― | ― |
| Military of Australia, 2009^**^ | MRDI | 17–30 | Low physical activity (1.5) | 920–2300 | 2.3–5.6 | 920–2300 | 2.3–5.6 |
|  |  |  | Moderately active (1.8) | 920–2500 | 2.3–6.1 | 920–2500 | 2.3–6.1 |
|  |  |  | Very active (2.1) | 920–2750 | 2.3–6.7 | 920–2750 | 2.3–6.7 |
|  |  |  | Extreme activity (2.4) | 920–3000 | 2.3–7.4 | 920–3000 | 2.3–7.4 |
|  |  |  | Believed to approach the limit of human endurance (3.2)^**^ | 920–3200 | 2.3–7.8 | ― | ― |
|  |  |  | unacclimatised people working in hot climates | <4600 | <11.3 | <4600 | <11.3 |
| Civilian of UK, 1991^#^ | LRNI | 15–50 | ― | 575 | 1.4 | 575 | 1.4 |
|  |  | 50 < | ― | 575 | 1.4 | 575 | 1.4 |
|  | RNI | 15–50 | ― | 1600 | 3.9 | 1600 | 3.9 |
|  |  | 50 < | ― | 1600 | 3.9 | 1600 | 3.9 |
| Civilian of USA, 2005, 2019^+^ | AI | 14–18 | ― | 1500 | 3.7 | 1500 | 3.7 |
|  |  | 19–70 | ― | 1500 | 3.7 | 1500 | 3.7 |
|  | CDRR | 14–18 | ― | 2300 | 5.6 | 2300 | 5.6 |
|  |  | 19–70 | ― | 2300 | 5.6 | 2300 | 5.6 |
| Military of USA, 1985 | MRDA | 17–50 | ― | ― | ― | ― | ― |
| Military of USA, 2001 | MDRI | 17–50 | ― | 5000  (4550–5525) | 12.3  (11.1–13.5) | 3600  (3220–3910) | 8.8  (7.9–9.6) |
| Military of USA, 2017 | MDRI | 19–30 | ― | 2300 | 5.6 | 2300 | 5.6 |
| NATO, 2010^‡^ | AI | 19–50 | Civilian norm (1.6) | 920 | 2.3 | ― | ― |
|  |  |  | Normal operations (2.0) | 920 | 2.3 | ― | ― |
|  |  |  | Combat or special forces operations (2.4) | 920 ^+1200–4800^ | 2.3^+2.9–11.8^ | ― | ― |
|  |  |  | Hot environment | 920 ^+1200–4800^ | 2.3^+2.9–11.8^ | ― | ― |
|  |  |  | Cold environment | 920 ^+1200–4800^ | 2.3^+2.9–11.8^ | ― | ― |
|  |  |  | High–altitude environment | 920 ^+1200–4800^ | 2.3^+2.9–11.8^ | ― | ― |
| The targets were healthy adults, excluding pregnant women and lactating women, but the criteria for the age range was organized to include 16 years old and over and under 65 years old, including 16 years old, which was the minimum age in the military report.  As in the Standard tables of food composition in Japan 2020 (eighth revised version), the salt equivalent (g) was calculated by multiplying sodium (mg) by 2.54.  ^*^NRV was revised in 2017 (SDT and UL for adults have been revised since 2006, with SDT 1600 mg/day→2000 mg/day and UL 2300 mg/day→not set.).  ^**^Because it is "believed to approach the limit of human endurance," it can be applied to male personnel responsible for special military activities. Therefore, category V is set only for adult males. In addition, for military personnel who are not accustomed to hot environments, when carrying out military activities in hot environments, the upper limit of intake was recommended to be 4,600 mg.  ^#^No formulation of DRI for military operations has been made. ^+^The DRI was revised in 2019 (In 2005, it was AI 14–50 years 1500 mg, 51–70 years 1300 mg, UL 19–50≦years 2300 mg).  ^‡^It was recommended to supplement between 1200 mg and 4800 mg, depending on the amount of sweat.  Abbreviation:  AI, Adequate Intake; CDRR, Chronic Disease Risk Reduction Intake; EAR, Estimated Average Requirement; LRNI, Lower Reference Nutrient Intake; MRDA, Military Recommended Dietary Allowances; MRDI, Military Recommended Dietary Intake; RNI, Reference Nutrient Intake; SDT, Suggested Dietary Target; UL, Upper Level of Intake | | | | | | | |

**Table S15. Iron in Dietary Reference Intakes (DRI) for civilians and military (mg/day)**

| DRI | Age (years) | Category (PAL) | Males | | | | | Females | | | | |
| --- | --- | --- | --- | --- | --- | --- | --- | --- | --- | --- | --- | --- |
|  |  |  | EAR | RDA | UL | Other | | EAR | RDA | UL | Other | |
| Civilian of Australia,　2006 | 14–18 | ー | 8.0 | 11.0 | 45.0 | ー | ー | 8.0 | 15.0 | 45.0 | ー | ー |
|  | 19–70 | ー | 6.0 | 8.0 | 45.0 | ー | ー | 8.0 | 18.0 | 45.0 | ー | ー |
|  |  | ー |  |  |  | ー | ー | 5.0 | 8.0 |  | ー | ー |
| Military of Australia, 2006 | 17–18 | ー | ー | 11.0 | ー | ー | ー | ー | 15.0 | ー | ー | ー |
|  | 19–30 | ー | ー | 8.0 | ー | ー | ー | ー | 18.0 | ー | ー | ー |
| Civilian of UK, 2011^#^ | 15–18 | ー | 8.7 | 11.3 | ー | LRNI | 6.1 | 11.4 | 14.8 | ー | LRNI | 8.0 |
|  | 19–50 | ー | 6.7 | 8.7 | ー |  | 4.7 | 11.4 | 14.8 | ー |  | 8.0 |
|  | 50 < | ー | 6.7 | 8.7 | ー |  | 4.7 | 6.7 | 8.7 | ー |  | 4.7 |
| Civilian of USA, 2001 | 14–18 | ー | 7.7 | 11.0 | 45.0 | ー | ー | 7.9 | 15.0 | 45.0 | ー | ー |
|  | 19–50 | ー | 6.0 | 8.0 | 45.0 | ー | ー | 8.1 | 18.0 | 45.0 | ー | ー |
|  | 51–70 | ー |  |  |  | ー | ー | 5.0 | 8.0 |  | ー | ー |
| Military of USA, 1985^+^ | 19–50 | ー | ー | 10-18 | ー | ー | ー | ー | 18.0 | ー | ー | ー |
| Military of USA, 2001 | 19–50 | ー | ー | 10.0 | ー | ー | ー | ー | 15.0 | ー | ー | ー |
| Military of USA, 2017 | 19–30 | ー | ー | 8.0 | ー | ー | ー | ー | 18.0 | ー | ー | ー |
| NATO, 2010 | 19–50 | Civilian norm (1.6) | ー | 8.0 | ー | ー | ー | ー | ー | ー | ー | ー |
|  |  | Normal operations (2.0) | ー | 8.0 | ー | ー | ー | ー | ー | ー | ー | ー |
|  |  | Combat or special forces operations (2.4) | ー | 14.0 | ー | ー | ー | ー | ー | ー | ー | ー |
|  |  | Hot environment | ー | 14.0 | ー | ー | ー | ー | ー | ー | ー | ー |
|  |  | Cold environment | ー | 15.0 | ー | ー | ー | ー | ー | ー | ー | ー |
|  |  | High–altitude environment | ー | 15.0 | ー | ー | ー | ー | ー | ー | ー | ー |
| The targets were healthy adults, excluding pregnant women and lactating women, but the criteria for the age range was organized to include 16 years old and over and under 65 years old, including 16 years old, which was the minimum age in the military report.  ^#^No formulation of DRI for military operations has been made. In addition, it was formulated after excluding female with high menstrual losses.  ^+^Higher requirements were reflected in military personnel aged 17–18 years than in adults (Vitamin D, Calcium, Phosphorus, Magnesium and Iron).    Abbreviation:  EAR, Estimated Average Requirement; LRNI, Lower Reference Nutrient Intake; RDA, Recommended Dietary Allowance; UL, Tolerable upper intake level | | | | | | | | | | | | |

**Table S16. Zinc in Dietary Reference Intakes (DRI) for civilians and military (mg/day)**

| DRI | Age (years) | Category (PAL) | Males | | | | | Females | | | | |  |  |
| --- | --- | --- | --- | --- | --- | --- | --- | --- | --- | --- | --- | --- | --- | --- |
|  |  |  | EAR | RDA | UL | Other | | EAR | RDA | UL | Other | |  |  |
| Civilian of Australia,　2006 | 14–18 | ー | 11 | 13 | 35 | ー | ー | 6.0 | 7.0 | 35 | ー | ー |  |  |
|  | 19–70 | ー | 12 | 14 | 40 | ー | ー | 6.5 | 8.0 | 40 | ー | ー |  |  |
| Military of Australia, 2006 | 17–18 | ー | ー | 13 | ー | ー | ー | ー | 7.0 | ー | ー | ー |  |  |
|  | 19–30 | ー | ー | 14 | ー | ー | ー | ー | 8.0 | ー | ー | ー |  |  |
| Civilian of UK, 2011^#^ | 15–18 | ー | 7.3 | 9.5 | ー | LRNI | 5.5 | 5.5 | 7.0 | ー | LRNI | 4.0 |  |  |
|  | 19–50 | ー | 7.3 | 9.5 | ー |  | 5.5 | 5.5 | 7.0 | ー |  | 4.0 |  |  |
|  | 50 < | ー | 7.3 | 9.5 | ー |  | 5.5 | 5.5 | 7.0 | ー |  | 4.0 |  |  |
| Civilian of USA, 2001 | 14–18 | ー | 8.5 | 11 | 34 | ー | ー | 7.3 | 9.0 | 34 | ー | ー |  |  |
|  | 19–70 | ー | 9.4 | 11 | 40 | ー | ー | 6.8 | 8.0 | 40 | ー | ー |  |  |
| Military of USA, 1985 | 17–50 | ー | ー | 15 | ー | ー | ー | ー | 15 | ー | ー | ー |  |  |
| Military of USA, 2001 | 17–50 | ー | ー | 15 | ー | ー | ー | ー | 12 | ー | ー | ー |  |  |
| Military of USA, 2017 | 19–30 | ー | ー | 11 | ー | ー | ー | ー | 8.0 | ー | ー | ー |  |  |
| NATO, 2010 | 19–50 | Civilian norm (1.6) | ー | 14 | ー | ー | ー | ー | ー | ー | ー | ー | |  |
|  |  | Normal operations (2.0) | ー | 14 | ー | ー | ー | ー | ー | ー | ー | ー | |  |
|  |  | Combat or special forces operations (2.4) | ー | 15 | ー | ー | ー | ー | ー | ー | ー | ー | |  |
|  |  | Hot environment | ー | 15 | ー | ー | ー | ー | ー | ー | ー | ー | |  |
|  |  | Cold environment | ー | 20 | ー | ー | ー | ー | ー | ー | ー | ー | |  |
|  |  | High–altitude environment | ー | 20 | ー | ー | ー | ー | ー | ー | ー | ー | |  |
| The targets were healthy adults, excluding pregnant women and lactating women, but the criteria for the age range was organized to include 16 years old and over and under 65 years old, including 16 years old, which was the minimum age in the military report.  ^#^No formulation of DRI for military operations has been made.    Abbreviation:  EAR, Estimated average requirement; RDA, Recommended Dietary Allowance; UL, Tolerable upper intake level; LRNI, Lower Reference Nutrient Intake | | | | | | | | | | | | | | |

**Table S17. Copper in Dietary Reference Intakes (DRI) for civilians and military (mg/day)**

| DRI | Age (years) | Category (PAL) | Males | | | | Females | | | |  |
| --- | --- | --- | --- | --- | --- | --- | --- | --- | --- | --- | --- |
|  |  |  | EAR | RDA | AI | UL | EAR | RDA | AI | UL |  |
| Civilian of Australia, 2006 | 14–18 | ー | ー | ー | 1.50 | 8 | ー | ー | 1.10 | 8 |  |
|  | 19–70 | ー | ー | ー | 1.70 | 10 | ー | ー | 1.20 | 10 |  |
| Military of Australia, 2009 | 17–18 | ー | ー | ー | 1.50 | ー | ー | ー | 1.10 | ー |  |
|  | 19–30 | ー | ー | ー | 1.70 | ー | ー | ー | 1.20 | ー |  |
| Civilian of UK, 2011^#^ | 15–18 | ー | 1.00 | ー | ー | ー | 1.00 | ー | ー | ー |  |
|  | 19–50 | ー | 1.20 | ー | ー | ー | 1.20 | ー | ー | ー |  |
|  | 50 < | ー | 1.20 | ー | ー | ー | 1.20 | ー | ー | ー |  |
| Civilian of USA, 2001^#^ | 14–18 | ー | 0.69 | 0.89 | ー | 8 | 0.69 | 0.89 | ー | 8 |  |
|  | 19–70 | ー | 0.70 | 0.90 | ー | 10 | 0.70 | 0.90 | ー | 10 |  |
| NATO, 2010 | 19–50 | Civilian norm (1.6) | ー | ー | 1.70 | ー | ー | ー | ー | ー |  |
|  |  | Normal operations (2.0) | ー | ー | 1.70 | ー | ー | ー | ー | ー |  |
|  |  | Combat or special forces operations (2.4) | ー | ー | 1.80 | ー | ー | ー | ー | ー |  |
|  |  | Hot environment | ー | ー | 1.80 | ー | ー | ー | ー | ー |  |
|  |  | Cold environment | ー | ー | 1.70 | ー | ー | ー | ー | ー |  |
|  |  | High–altitude environment | ー | ー | 1.70 | ー | ー | ー | ー | ー |  |
| The targets were healthy adults, excluding pregnant women and lactating women, but the criteria for the age range was organized to include 16 years old and over and under 65 years old, including 16 years old, which was the minimum age in the military report.  ^#^No formulation of DRI for military operations has been made.    Abbreviation:  EAR, Estimated average requirement; RDA, Recommended Dietary Allowance; UL, Tolerable upper intake level; LRNI, Lower Reference Nutrient Intake | | | | | | | | | | | |

**Table S18. Macronutrient energy istribution in Dietary Reference Intakes (DRI) for military with the addition of Singapore, China, and South Korea (/day)**

| DRI | Age (years) | Category (PAL) | Males | | | | | | |
| --- | --- | --- | --- | --- | --- | --- | --- | --- | --- |
|  |  |  | Energy | Protein | | Fat | | Carbohydrate | |
|  |  |  | kcal | g | %E | g | %E | g | %E |
| Military of Australia, 2009 | 17–18 | Low physical activity (1.5) | 2988 | 110–147 | 15–20 | ― | 25–35 | 266–292 | 50–55 |
|  |  | Moderately active (1.8) | 3466 | 119–162 | 14–19 | ― | 24–34 | 325–356 | 52–57 |
|  |  | Very active (2.1) | 3944 | 126–175 | 13–18 | ― | 23–33 | 388–424 | 54–59 |
|  |  | Extreme activity (2.4) | 4661 | 138–195 | 12–17 | ― | 22–32 | 455–496 | 56–61 |
|  | 19–30 | Low physical activity (1.5) | 2868 | 106–141 | 15–20 | ― | 25–35 | 375–413 | 50–55 |
|  |  | Moderately active (1.8) | 3346 | 115–156 | 14–19 | ― | 24–34 | 455–499 | 52–57 |
|  |  | Very active (2.1) | 3824 | 122–169 | 13–18 | ― | 23–33 | 540–590 | 54–59 |
|  |  | Extreme activity (2.4) | 4541 | 134–190 | 12–17 | ― | 22–32 | 665–724 | 56–61 |
|  |  | Believed to approach the limit of human endurance (3.2)^**^ | 5975 | 162–235 | 11–16 | ― | 21–31 | 906–984 | 58–63 |
|  | ― | High–altitude environment ^***^ | ― | ― | 15 | ― | 20 | ― | 65 |
| Military of UK, 2016, 2020^#^ | 16–60 | General population (1.6) | 2900 | ― | 15 | ― | 35 | ― | 50 |
|  |  | Active service (2.1) | 3600 | ― | 13.5–15 | ― | 31.5–35 | ― | 50–55 |
|  |  | Military training courses A (2.3) | 4100 | ― | 12–15 | ― | 28–35 | ― | 50–60 |
|  |  | Military training courses B (2.6) | 4600 | ― | 10–15 | ― | 25–35 | ― | 50-65 |
| Military of USA, 2017^+^ | 19–30 | General population (1.6)^+^ | 3400 | 102 (68–136) | 10–35 | <113 (100–157) | <30 | 510 (340–680) | 50–55 |
|  |  | Active service (2.1) | 3000 |  |  |  |  |  |  |
|  |  | Military training courses A (2.3) | 3400 |  |  |  |  |  |  |
|  |  | Military training courses B (2.6) | 3700 |  |  |  |  |  |  |
|  |  | General population (1.6) | 4700 |  |  |  |  |  |  |
| NATO, 2010^‡^ | 19–50 | Civilian norm (1.6) | 2900 | 144  (108–179) | 15–25 | 88 (64–111) | 20–35 | 395 (323–466) | 45–65 |
|  |  | Normal operations (2.0) | 3600 | 180 (135–225) |  | 110 (80–140) |  | 494 (404–584) |  |
|  |  | Combat or special forces operations (2.4) | 4900 | 246 (184–307) |  | 150 (109–191) |  | 675 (552–797) |  |
|  |  | Hot environment | 4900 | 246 (184–307) |  | 150 (109–191) |  | 675 (552–797) |  |
|  |  | Cold environment | 4900 | 246 (184–307) |  | 150 (109–191) |  | 675 (552–797) |  |
|  |  | High–altitude environment | 4700 | 178 (178–296) | 15 | 132  (105–184) | ≦25 | 711 (533–770) | 60 |
| Singapore, 2016 | 18–30 | Normal (1.47) | 2500 | 94 | ― | <76 | ― | 359 | ― |
|  |  | Active (1.95) | 3000 | 95 |  | <81 |  | 473 |  |
|  |  | High active (2.27) | 3500 | 96 |  | <97 |  | 560 |  |
|  |  | Very high active (2.59) | 4000 | 105 |  | <111 |  | 654 |  |
| China, 2016 | 18–30 | Army / Low physical activity | 2600–< 3000 | 90 | ― | ― | ― | ― | ― |
|  |  | Army / Moderately active | 3000–< 3500 | 100 |  |  |  |  |  |
|  |  | Army / Very active | 3500–< 4000 | 120 |  |  |  |  |  |
|  |  | Army / Extreme activity | 4000–< 4500 | 130 |  |  |  |  |  |
|  |  | Navy / Surface ship | 3300–< 3600 | 110 |  |  |  |  |  |
|  |  | Navy / Submarine | 3300–< 3600 | 120 |  |  |  |  |  |
|  |  | Navy / Nuclear submarine | 3500–< 3700 | 120.0 |  |  |  |  |  |
|  |  | Air Force / Pilot | 3100–< 3600 | 120.0 |  |  |  |  |  |
| Korea, 2017 | 19–29 | ― | 3000 | 127.5 | 17.0 | 74 | 22.2 | 456 | 60.8 |
| The targets were healthy adult males, but the criteria for the age range was organized to include 16 years old and over and under 65 years old, including 16 years old, which was the minimum age in the military report. | | | | | | | | | |
